# Towards on-chip time-resolved thermal mapping with micro-/nanosensor arrays

**DOI:** 10.1186/1556-276X-7-484

**Published:** 2012-08-29

**Authors:** Haixiao Liu, Weiqiang Sun, An Xiang, Tuanwei Shi, Qing Chen, Shengyong Xu

**Affiliations:** 1 Key Laboratory for the Physics and Chemistry of Nanodevices, Peking University, Beijing, 100871, China; 2 Department of Electronics, Peking University, Beijing, 100871, China; 3 Academy for Advanced Interdisciplinary Studies, Peking University, Beijing, 100871, China; 4 National Center for Nanoscience and Technology, Beijing, 100190, China

**Keywords:** Real-time thermal mapping, Sensor array, Thin-film thermocouple, Nanoscale thermometry, On-chip hot spot detection

## Abstract

In recent years, thin-film thermocouple (TFTC) array emerged as a versatile candidate in micro-/nanoscale local temperature sensing for its high resolution, passive working mode, and easy fabrication. However, some key issues need to be taken into consideration before real instrumentation and industrial applications of TFTC array. In this work, we will demonstrate that TFTC array can be highly scalable from micrometers to nanometers and that there are potential applications of TFTC array in integrated circuits, including time-resolvable two-dimensional thermal mapping and tracing the heat source of a device. Some potential problems and relevant solutions from a view of industrial applications will be discussed in terms of material selection, multiplexer reading, pattern designing, and cold-junction compensation. We show that the TFTC array is a powerful tool for research fields such as chip thermal management, lab-on-a-chip, and other novel electrical, optical, or thermal devices.

## Background

One of the major bottlenecks of fast development in the modern electronics industry is heat. Managing the high heat flux generated by billions of transistors in an integrated circuit (IC) is the main challenge. A scalable, built-in, and agile temperature sensor which can provide real-time thermal history of an IC chip is essential to thermal failure analysis [1]. However, thermal measurement at microscale and nanoscale is a technical challenge. When both high spatial resolution and high temperature sensitivity are required, only a few approaches, such as scanning thermal microscopy [2], luminescent nanoparticles, and infrared thermography [3-5], remain in the list of suitable candidates. If built-in thermal sensors are required for time-resolvable solutions in a solid device, such as in IC chips, micro-electro-mechanical system, a lab-on-a-chip, or a flexible printed circuits, then it seems that only stripe-shaped resistive sensor [6] and thin-film thermocouple (TFTC) are left in the candidate list. The passive nature of a thermocouple makes it superior to a resistive thermal sensor (usually made of metal or semiconductors) because a thermocouple provides a far more rapid response, does not require external excitation voltage or current, and generates no additional heat which may cause unexpected electrical noise and error in temperature measurement.

A TFTC works on a well-known Seebeck effect [7], i.e., a thermoelectric voltage will be generated when a temperature difference is established between two ends of a conductor. The ability to generate thermoelectric signal was symbolized by the Seebeck coefficient (*S*). A TFTC is made when two different conductors *α* and *β* join together at one end (*hot end*). If there is a temperature difference (Δ*T*), then a thermoelectric voltage (Δ*V*) can be collected at the open end (usually kept cold, referred as the *cold end*) as follows:

(1) $$\Delta V=\left(S_{\alpha }-S_{\beta }\right)\cdot \left(T_{\text{h}}-T_{\text{c}}\right)=\text{TP}\cdot \Delta T\text{,}$$

where *S*_*α*_ and *S*_*β*_ represent the Seebeck coefficients of *α* and *β*, and *T*_h_ and *T*_c_ are the temperatures at the *hot end* and *cold end*, respectively. TP means the thermopower of thermocouple, which is the sensitivity of the sensor. Given the measured Δ*VT*_c_, and TP, one can easily calculate the *T*_h_. By embedding thermocouples in an electrical device, e.g., an IC chip, one can monitor the temperature at the area of thermocouple junction even after packaging. In addition, the measured voltage signal is just relative to the temperature of the junction area; thus, by shrinking the junction area of the thermocouple, one can obtain a highly localized temperature measurement. Pioneering work on scaling down the size of a TFTC has pushed its junction area to 100 × 100 nm [8], suggesting the potential for local temperature sensing at the nanoscale. Finally, the fabrication process for a TFTC is compatible with the well-developed microelectronic techniques. As a result, TFTCs have attracted much attention in recent years [9-15].

In 2005, Park et al. firstly constructed 100 TFTCs into an array providing two-dimensional (2D) temperature mapping in an area of around a square centimeter [12]. A few years later, Grayson et al. discussed some considerations in the progress of instrument TFTC array into real IC chips [13,14]. Recently, we presented a novel method for time-resolved 2D mapping by TFTC array [15] and firstly constructed a functional TFTC array made of a single material [16]. As a built-in solution, TFTC array is gaining more and more attention. However, to further push forward the development of TFTC array and its instrumentation in electronics industry, more potential advantages have to be explored, and some related issues have to be addressed.

In this work, we report on our recent work on TFTC array, including construction of the first nanoscale TFTC array in an area of 5 × 5 μm. We demonstrate that besides real-time temperature mapping, the TFTC array can also be utilized in mapping time-resolved heat flux and tracing a possible hot spot on a surface. We also discuss some key issues of instrumentation in IC chips in the future, including choice of material, multiplexing, pattern designing, and *cold end* problems.

## Methods

### Material selection

Semiconductor-based thermocouples play an important role in industries on thermal sensing because of their high Seebeck coefficients, which are usually 20 to 500 times higher than metal-based thermocouples. Thus, thermocouples based on silicon, germanium, tellurium, or semiconductor compounds were often adopted in occasions that required high sensitivity; see Table 1[17,18]. Application-specific chips like infrared radiation detection were frequently reported [19-21], where high dependency between output signal and the heat induced by incident infrared radiation was preferable. The semiconductor-based thermocouples were connected in series as a thermopile to provide larger output signals, typically 10 V/W.

**Table 1:** Typical physical properties and figure of merits of candidate materials (300 K)

**Materials**	***S*(μV/K)**	***ρ*(Ωm)**	***κ*(W/m K)**	**ZT**
Cr	18.8	1.27E − 7	93.9	8.9E − 3
Ni	−18	7E − 8	90.9	1.5E − 2
Al	−1.7	2.65E − 8	237	1.4E − 4
Si^a^	Approximately −450	1.4	132	3.3E − 7
poly-Si^a^	Approximately −1,200	0.36	135	8.9E − 6
n-poly-Si^b^	−57	8.1E − 6	31.5	3.8E − 3
p-poly-Si^b^	103	2.2E − 5	31.2	4.6E − 3
n-poly-SiGe^b^	−77	2.4E − 5	9.4	7.9E − 3
p-poly-SiGe^b^	59	1.9E − 5	11.1	5.0E − 3

^a^The samples of Si and poly-Si have intrinsic N-type impurities [17]; ^b^the doping concentration was 2.5E20 atoms/cm^3^[18].

**Figure 1:**
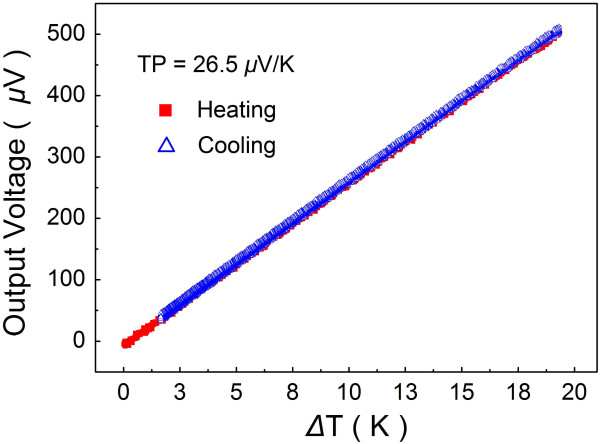
**A typical 100-nm-thick Cr-Ni TFTC calibration data.** The TP is calculated from the slope of the plot.

However, in ordinary temperature measuring situations, semiconductor-based TFTCs were not selected extensively. From Table 1, we can find very low figure of merit (ZT) in all these materials, which means that they can hardly be ideal materials in thermoelectric applications like cooling or power generation with high efficiency. If *n*-type and *p*-type materials with high figure of merit (ZT ≈ 3) can really be synthesized in the future, thermoelectric pairs made by these materials can probably function as chip coolers or micro-power generators with the temperature measuring at the same time. As only for thermal measurement, the Seebeck coefficient is our main concern, and the figure of merit is not so critical. We choose Cr and Ni as TFTC composition materials. The Cr-Ni TFTCs 100-nm thick provide TP of around 26.2 ± 1.5 μV/K stably [15], which is large enough to provide a temperature resolution better than 0.1 K. Typical Cr-Ni TFTC characterization data are presented in Figure 1, where the negligible hysteresis in heating and cooling processes indicates the reliability.

Moreover, unlike the intrinsic Seebeck coefficient of metals, the Seebeck coefficient of semiconductor is highly extrinsic in relevance with the doping density, crystal structure, and resistivity. For example, the Seebeck coefficient of *p*-type silicon varied from 300 to 1,600 μV/K at room temperature [22]. Because of its relevance to fabrication process, the thermopower of semiconductor-based thermocouple may vary from batch to batch or even from one to another, which was hard to put into industrial batch productions. As for the metal-based thermocouples, they were put into wide applications for many years, and industrial standard thermocouples were all made of metals or alloys. Thin-film metal thermocouple can generate a similar thermopower given similar film thicknesses. Varrenti et al. first reported the reproducibility of Cr-Ni TFTCs, both inter- and intra-batch [14]. Our work also contributed to the high stability of Cr-Ni TFTCs by massive calibrations [15].

As to the fabrication, Cr-Ni TFTCs can also be produced in CMOS-compatible processes just like the silicon-based temperature sensors. The differences were that sputtering was used in metal fabrications, and diffusion or ion implantation was adopted in doped silicon stripe fabrication. Si-based thermocouple fabrication may require additional lithography and etching to remove the superfluous undoped silicon to avoid short circuit. Besides, metals can be incorporated in different layers of ICs. The silicon-based device always synthesized in the same layer of the ICs, consuming precious spaces.

### Array designing and multiplexing

**Figure 2:**
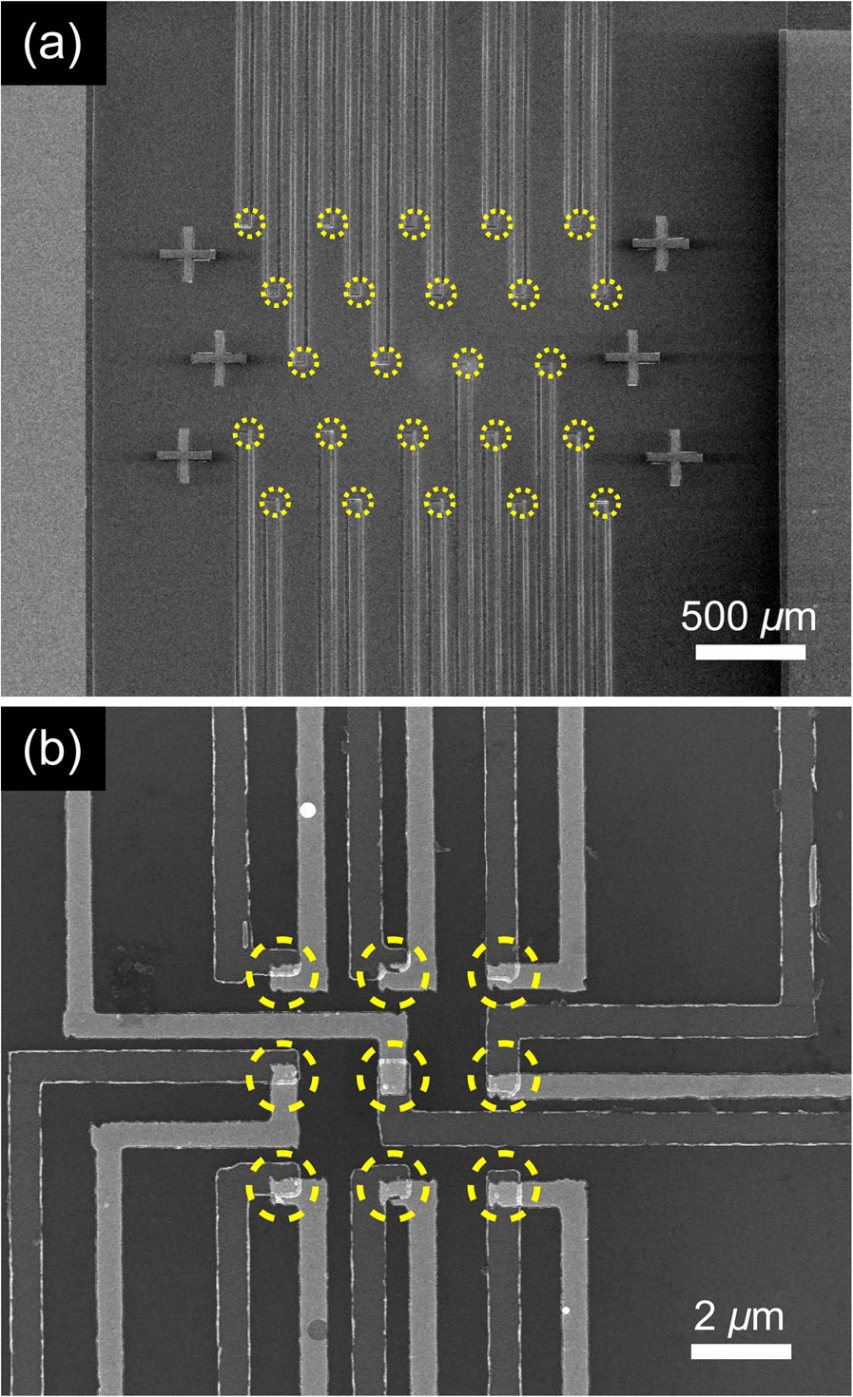
**SEM micrograph of micro-/nanoscale TFTC array. (a)** A TFTC array with 24 TFTCs in an area of around 1 × 1 mm. **(b)** A 3 × 3 TFTC array in an area of 5 × 5 μm, with a critical size of 500 nm.

Previously reported TFTC arrays were all constructed in an area larger than 5 × 5 mm [12,13,15]; we show that the size, density, and pattern of a TFTC array can be far more flexible. Using standard industrial processes of photo lithography, e-beam lithography, and thin film deposition, we have made micro-/nanoscale TFTC arrays in 3 × 3, 3 × 6, 5 × 5, 8 × 8, and 10 × 10 matrices from an area of 1 × 2 cm to an area of 5 × 5 μm. As a illustration, Figure 2 shows TFTC arrays with critical sizes of 20 μm and 500 nm, respectively. The TFTC array in Figure 2a dwells in an area of 1 × 1.2 mm, while another TFTC array in Figure 2b is around 5 × 5 μm, which is, to the best of our knowledge, the smallest TFTC array ever been made. They can provide precise local temperature mapping in highly scalable resolutions.

As to on-chip applications, a high surface density of TFTCs certainly increases the spatial resolution and accuracy in temperature measurement, but it also remarkably increases the number of pads and connections for the TFTC array, leading to complex interconnection. Therefore, the density of sensors in the array should be balanced between being enough to give required sensing resolution and being minimum to reduce the complexity of interconnection and space of pads.

We have found that matrices with relatively high densities were not necessary in integrated circuit temperature measurement since the ‘hot spot’ in a chip will provide temperature at least 20 times higher than the ambient temperature. Matrices of 3 × 3 or 3 × 6 TFTC array provided a temperature mapping good enough to help in judging where the hot spot was and how the thermal field time-dependently changed.

In addition, the density of TFTC array may have an influence on the real-time monitoring of local temperatures. Usually, one uses only one voltmeter to subsequently measure all the thermocouples in one array, with the help of a multiplexer. However, since a reliable reading process by a voltmeter needs a minimum time of 1 to 10 ms, if the total number of sensors in a TFTC array is too large, then one cycle of the measurements which ensures each individual sensor is measured once may take too long; thus, a real-time monitoring of the 2D temperature map may not be practical.

## Results and discussion

### Thermal 2D mapping

**Figure 3:**
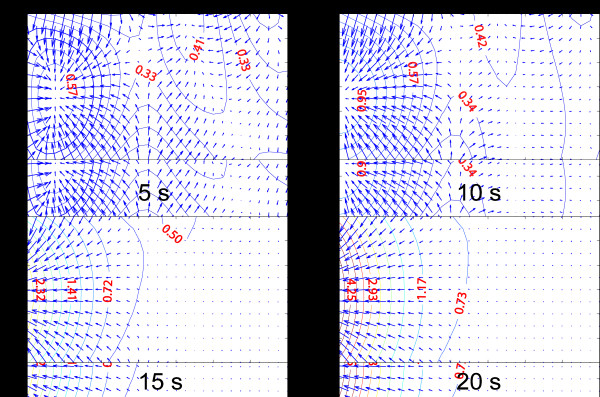
**Time-resolved temperature and heat flux mapping data with slowly heated heat source located left side.** The red text indicates the temperature rise in the isothermal lines. Along with the slow heating, the heat flux moved to indicate the direction of the heat source from the 10 s.

Using multiplexing with high sampling rate in an array, one can easily obtain thermal readings of each TFTC in sequence. Given the heat that usually flows slowly and smoothly, an interpolation method can be applied to obtain the thermal mapping in different time windows. We have shown a multimedia movie to illustrate time-resolved temperature mapping by a TFTC array [15]. Moreover, more thermal information can be revealed by a simple built-in TFTC array for on-chip thermal analysis, which provided suitable array density and sampling rate. According to Fourier’s law, if the temperature field *T*(*x,*y*,*t) can be obtained reliably, then a time-resolved heat flux vector field $\vec{q}\left(x,\text{y},\text{t}\right)$ can be calculated directly from $\vec{q}\left(x,\text{y},\text{t}\right)=-\kappa \cdot \;\vec{\nabla }\;T\left(x,\text{y},\text{t}\right)$. In chip failure analysis, heat flux data are usually more important for engineers to improve their design [23]. In our study, when a heating process commerced at the left side of the 3 × 6 TFTC array, a real-time heat flux mapping data were obtained, as shown in Figure 3.

### Tracing the position of heat source

In on-chip applications, one cannot expect the hot spot to be always right on top of a TFTC. Unexpected failure in a working chip is always accompanied with unexpected hot spot induced. Tracing the possible locations of these unexpected hot spots is tricky for technicians. Inspired by a similar subject in signal processing, we propose to use a regular TFTC array to trace the heat.

**Figure 4:**
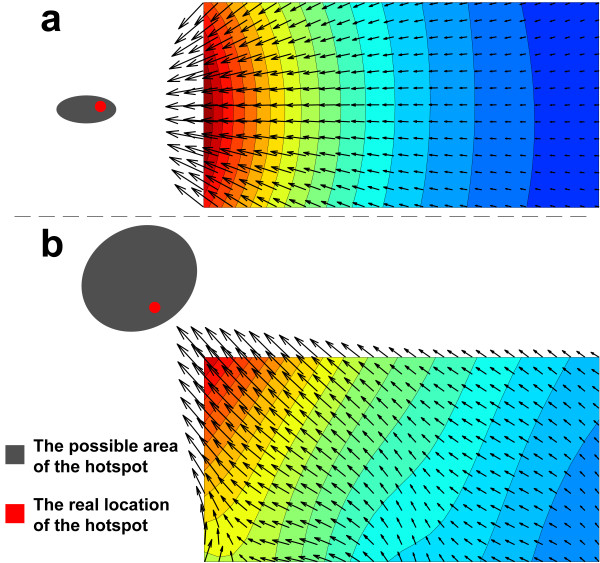
**Inverse tracing of the hot spot using a set of TFTC array. (a, b)** Two cases that show the capability of tracing the heating source from 2D maps of local temperatures. By proper calculations, one can figure out the temperature fields and the flow of the heat that transfer throughout the substrate surface. The arrows in the figure are plotted inversely following the gradient of temperatures in the maps. In each figure, the red dot is the exact location of the in-plane heater, while the grey-filled circle is the calculated location of heating source from the measured data.

In each contour line of a 2D map of local temperature field *T*(*x,*y*,*t), the temperature reads the same. From the heat flux field calculated from the gradient of *T*(*x,*y*,*t), we can even trace the heating source. By inversely tracing back the heat flow, one can find the source of the heat. Figure 4 shows two examples where a simple TFTC array of a 3 × 6 matrix is used to trace the heating source. The arrows in the figure are plotted inversely following the gradient of temperatures in the maps. In each figure, the red dot is the exact location of the in-plane heater, while the grey-filled circle is the calculated location of heating source from the measured data. They actually match pretty well. Further work will be put on improving the precision of the source tracing.

### Industrial on-chip applications

**Figure 5:**
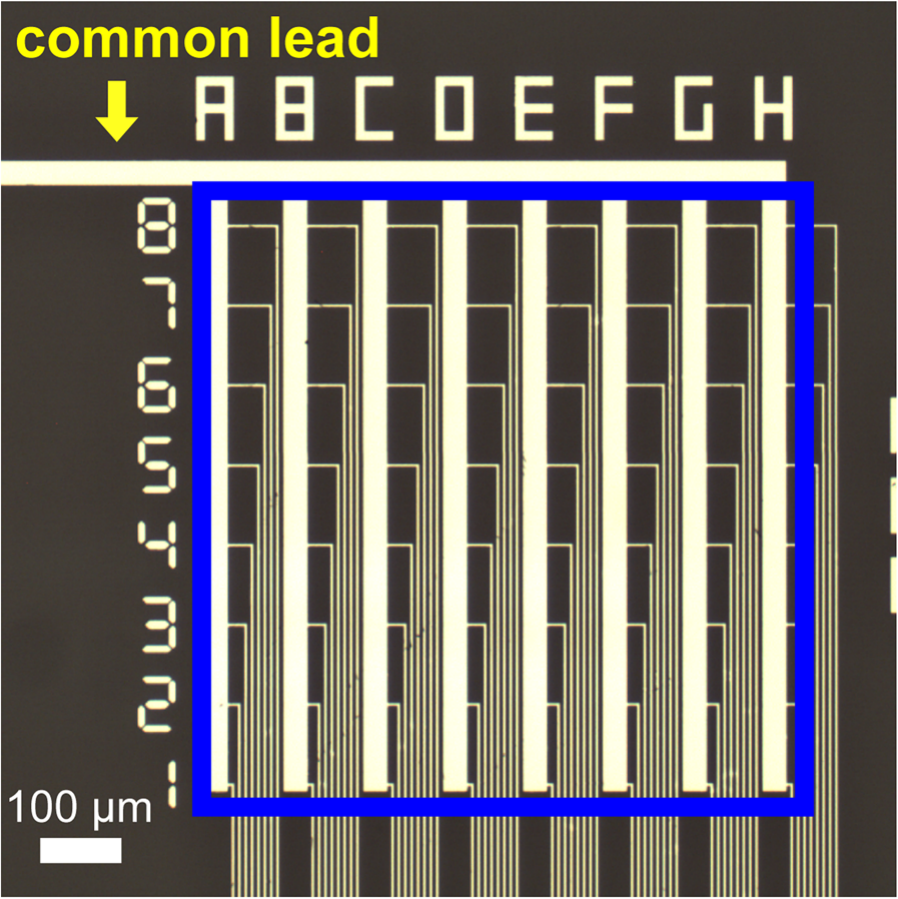
**Optical photograph of a TFTC array with a common lead configuration.** The array has 64 sensors arranged in an 8 × 8 square matrix, designed with a common lead configuration. The size of the array is 700 × 700 μm. Note that in the array, eight sensors in one series share one common lead, and all 64 sensors share one common bus line.

When putting the TFTC array in industrial applications, more possible problems need to be considered. One is that there are too many electrical pads induced with a TFTC array. A practical way to reduce the number of pads is to let a number of TFTCs share one common lead. By doing so, the pads and connections of TFTC array will shrink to nearly 50 % of the previous number. As the Cr-Ni thermocouple gives a relatively high thermopower, a common lead strategy can be adopted in designing without apparent error induced. Figure 5 shows an optical photograph of an 8 × 8 TFTC array that has the common lead configuration.

Another potential problem is that the temperature rises in the *cold ends* of TFTCs. In a TFTC built-in IC chip, because of the excellent thermal conductivity of silicon substrate when the IC circuit has been run for a long time, joule heat generated from the running current may heat up the entire chip, causing a remarkable rise of temperature at the *cold ends* of the TFTCs, thus causing a huge error in temperature readings. It seems that in this case, on-chip sensors of diodes or transistors can provide better temperature reading [24]. However, there are at least four defects which limit the development of these methods and why many endeavors were made in developing new ways of temperature sensing in IC chips.

Firstly, such methods all require active parts like electrical sources, which bring additional consumption and self-induced heat. Secondly, diode, transistors, and thermistor sensors often exhibit nonlinear temperature characteristics in operating temperature range, and their property may vary sensitively to the fabrication process, so calibrations will be often adopted to test the temperature characteristic. Offset was also usually programmed into the sensors to give additional compensation. Thirdly, the diode sensors cannot be placed just in the hottest area of the chip because of routing and I/O limitations [25]. In chips with elements of high density like the Intel® CoreTM Duo processor, the hot spots tend to vary from place to place according to different loaded watt power. Imprecision from 2°C to 10°C caused by non-linearity and hot spot drifting was not strange by adopting diode temperature sensors [25].

In contrast, although the inherent defects of thermocouple-based sensor can just measure the temperature differences spanned on the device, they have all the merits of having no active parts, no electrical sources, being simple and cheap, having linear responses, and being repeatable and quick enough for time-resolved profile. Possible solutions to minimize the impact of temperature of cold end were addressed as follows:

It is true that the difficulty of local temperature mapping by TFTC array increases as the chip area is scaling down, especially for very small silicon-based chips, e.g., 5 × 5 mm. It will not be easy to carry out on-chip thermal management based on the TFTC array. However, one of the properties of TFTCs is that their property will not be affected by the device routing, and the output signal of TFTCs is only contingent with temperature differences between the *hot ends* and the *cold ends*. Thus, we can arrange a common *cold end* for all the TFTCs. As they are sharing one *cold end*, we can still map the relative temperature differences and locate the hot spot. In most situations, IC chips which need thermal management will not be as small as 5 × 5 mm, and the hot spots are usually distributed locally in only parts of the working components of high density [25], so the distance between the junction area and the cold end can easily exceed 1 cm, and arranging the cold end on the fringe of the silicon die will not provide significant measurement error. A cooling base or cooling fan will soon dissipate the heat at the marginal area of the chip. We have produced micro- or nano-TFTC arrays on silicon substrates from 1 × 1 cm to 4 inch in diameter, with pads on the wafer edges. The TFTC array still functioned well by simply referring the temperature of the pads as room temperature.

## Conclusions

We have demonstrated micro- and nanoscale TFTC arrays as a powerful built-in thermal mapping tool. Using multiplexing technique, time-resolved temperature mapping can be easily drawn from TFTC array readings. Moreover, we have shown that TFTC array can also map real-time heat flux in plane, and the 2D mapping results can be used to precisely trace the heating source located in the surface.

Bearing in mind that the TFTC array can be used for on-chip solutions in the future, we discussed several remaining issues for its industrialization, i.e., choices of materials, density of sensor, sampling rate, common-lead strategy, and *cold end* temperature shift and solutions. We have demonstrated that a configuration of common lead can substantially reduce the number of pads by 50%.

We conclude that a variety of configuration of TFTC arrays is the most promising candidate as built-in sensors in 2D mapping of local temperature for solid devices such as IC chips because they are highly scalable from macro- to nanoscale, providing the potential of extremely high resolution and because they have a simple configuration, passive working mode, time-resolvable capability for real-time monitoring, and the capability for versatile applications.

## Competing interests

The authors declare that they have no competing interests.

## Authors’ contributions

HXL carried out most of the experiments on sample preparation and dynamic thermal mapping. WQS designed some sample patterns and contributed valuable discussions. AX and TWS conducted preparation of some samples with e-beam lithography and helped in data analysis. QC contributed on important experimental resources. SYX conceived the study and coordinated experiments. All authors read and approved the final manuscript.
